# Tumor-derived extracellular vesicles: key drivers of immunomodulation in breast cancer

**DOI:** 10.3389/fimmu.2025.1548535

**Published:** 2025-03-04

**Authors:** Jieming Li, Shuo Yu, Min Rao, Bomin Cheng

**Affiliations:** ^1^ Traditional Chinese Medicine (Zhong Jing) School, Henan University of Chinese Medicine, Zhengzhou, China; ^2^ Department of Polysaccharides and Drugs, Henan Key Laboratory of Chinese Medicine, Zhengzhou, China; ^3^ Department of Biliary-Pancreatic Surgery, Affiliated Tongji Hospital, Tongji Medical College, Huazhong University of Science and Technology, Wuhan, China; ^4^ Nursing Department, Tongji Hospital, Tongji Medical College, Huazhong University of Science and Technology, Wuhan, China; ^5^ Chinese Medicine Health Management Center, Shenzhen Traditional Chinese Medicine Hospital, Shenzhen, China

**Keywords:** breast cancer, extracellular vesicles, immune regulation, macrophages, T cells

## Abstract

Breast cancer (BC) remains a significant global health challenge characterized by its heterogeneity and treatment complexities. Extracellular vesicles (EVs) are small membranous particles released by cells, facilitating intercellular communication by transporting bioactive molecules such as proteins, lipids, and nucleic acids. Tumor-derived EVs have emerged as pivotal regulators in the tumor microenvironment (TME) and drivers of BC progression. These EVs carry diverse cargoes of bioactive molecules, influencing critical processes such as immune modulation, angiogenesis, and metastasis. By altering the behaviors of immune cells including macrophages, dendritic cells, and T cells, tumor-derived EVs contribute to immune evasion and tumor growth. Furthermore, Tumor-derived EVs play a role in mediating drug resistance, impacting the effectiveness of therapeutic interventions. Understanding the multifaceted roles of BC tumor-derived EVs is essential for the development of innovative therapeutic strategies. Targeting pathways mediated by EVs holds promise for enhancing the efficacy of cancer treatments and improving patient outcomes. This comprehensive review provides insights into the intricate interactions of tumor-derived EVs in immune modulation and BC progression, highlighting potential therapeutic targets and avenues for novel cancer therapies.

## 1 Introduction

Breast cancer (BC) remains one of the most prevalent and challenging malignancies worldwide, characterized by its heterogeneity and complex treatment responses (1). Despite advances in early detection and targeted therapies, BC continues to be a leading cause of cancer-related mortality among women (2). The intricacies of BC progression, including metastasis and resistance to therapy, necessitate a deeper understanding of the underlying mechanisms driving these processes (3).

Extracellular vesicles (EVs) are small, membrane-bound particles released by cells that play a crucial role in intercellular communication (4). EVs carry a diverse array of bioactive molecules such as proteins, lipids, and nucleic acids, facilitating the transfer of information between cells (5). In the context of cancer, EVs are increasingly recognized for their fundamental role in modulating the tumor microenvironment (TME), promoting tumor growth, and aiding in immune evasion (6). In BC, tumor-derived EVs are instrumental in reshaping the TME, contributing to processes such as angiogenesis, immune suppression, and the facilitation of metastasis (7). By transferring oncogenic factors and regulatory RNAs, these EVs can alter the behavior of recipient cells, promoting a pro-tumorigenic environment. For instance, EVs from HER-2-positive BC cells overexpressing Neuromedin U (NmU) transfer immunosuppressive cytokines, including TGFβ1 and PD-L1, to drug-sensitive cells (8). This transfer enhances immune evasion and confers resistance to trastuzumab-mediated cytotoxicity. EVs can induce the polarization of macrophages to a tumor-supportive phenotype and impair the function of immune cells like T cells and dendritic cells, thereby weakening anti-tumor response (9).

Therefore, we aim to explore the multifaceted roles of BC tumor-derived EVs in tumor progression and their potential as therapeutic targets based on recipient cells, as well as the limitations and potential perspectives. By understanding the mechanisms through which EVs modulate the TME and contribute to BC progression, we can develop novel strategies to enhance treatment efficacy. The insights gained could pave the way for innovative approaches in cancer therapy, ultimately improving patient outcomes and survival rates. It is nothing that the overarching term “extracellular vesicles (EVs)” is predominantly employed, and the terms “exosomes” and “microvesicles” are used in this article in accordance with their usage in the original studies cited.

## 2 EVs biogenesis and features

### 2.1 EVs biogenesis

EVs are heterogeneous populations of membrane-bound vesicles released by cells into the extracellular environment (10). EVs vesicles play pivotal roles in intercellular communication, influencing various physiological and pathological processes, including cancer progression (11).

The biogenesis of EVs encompasses multiple pathways, resulting in different types of vesicles, such as microvesicles and exosomes (12). Microvesicles are formed by the outward budding and fission of the plasma membrane, a process regulated by cytoskeletal components and signaling molecules like calcium and small GTPases (13, 14). Exosomes are small EVs ranging from 30 to 150 nanometers in diameter, originating from the endosomal compartment of cells (15). Exosome biogenesis begins with the inward budding of the cell membrane to form early endosomes, which mature into multivesicular bodies (MVBs) containing intraluminal vesicles (ILVs) (16). The formation of ILVs within MVBs is a critical step, driven by the endosomal sorting complexes required for transport (ESCRT) machinery. However, ESCRT-independent pathways also contribute to exosome formation, involving lipid raft microdomains and tetraspanins like CD63, CD81, and CD9. The fate of MVBs determines whether ILVs are degraded or secreted as exosomes. Fusion of MVBs with lysosomes leads to degradation, while fusion with the plasma membrane results in the release of exosomes into the extracellular space (17, 18). This release is regulated by Rab GTPases, particularly Rab27a and Rab27b, which are crucial for MVB docking and fusion with the cell membrane (19).

### 2.2 Exosome features within TME

EVs are distinguished by their unique lipid bilayer membranes enriched with molecules like cholesterol, sphingomyelin, and ceramide, which provide stability and protect their diverse cargoes (16, 20). These vesicles carry a variety of bioactive molecules, including proteins, lipids, and nucleic acids, reflecting the physiological state of their cell of origin and influencing their functional roles (21). Surface molecules such as tetraspanins (CD9, CD63, CD81) and major histocompatibility complex (MHC) proteins are key identifiers of EVs, facilitating cellular adhesion and immune modulation (22). Internally, EVs contain heat shock proteins (HSP70, HSP90) and nucleic acids like mRNAs and miRNAs, which regulate gene expression in recipient cells (23, 24).

**Figure 1:**
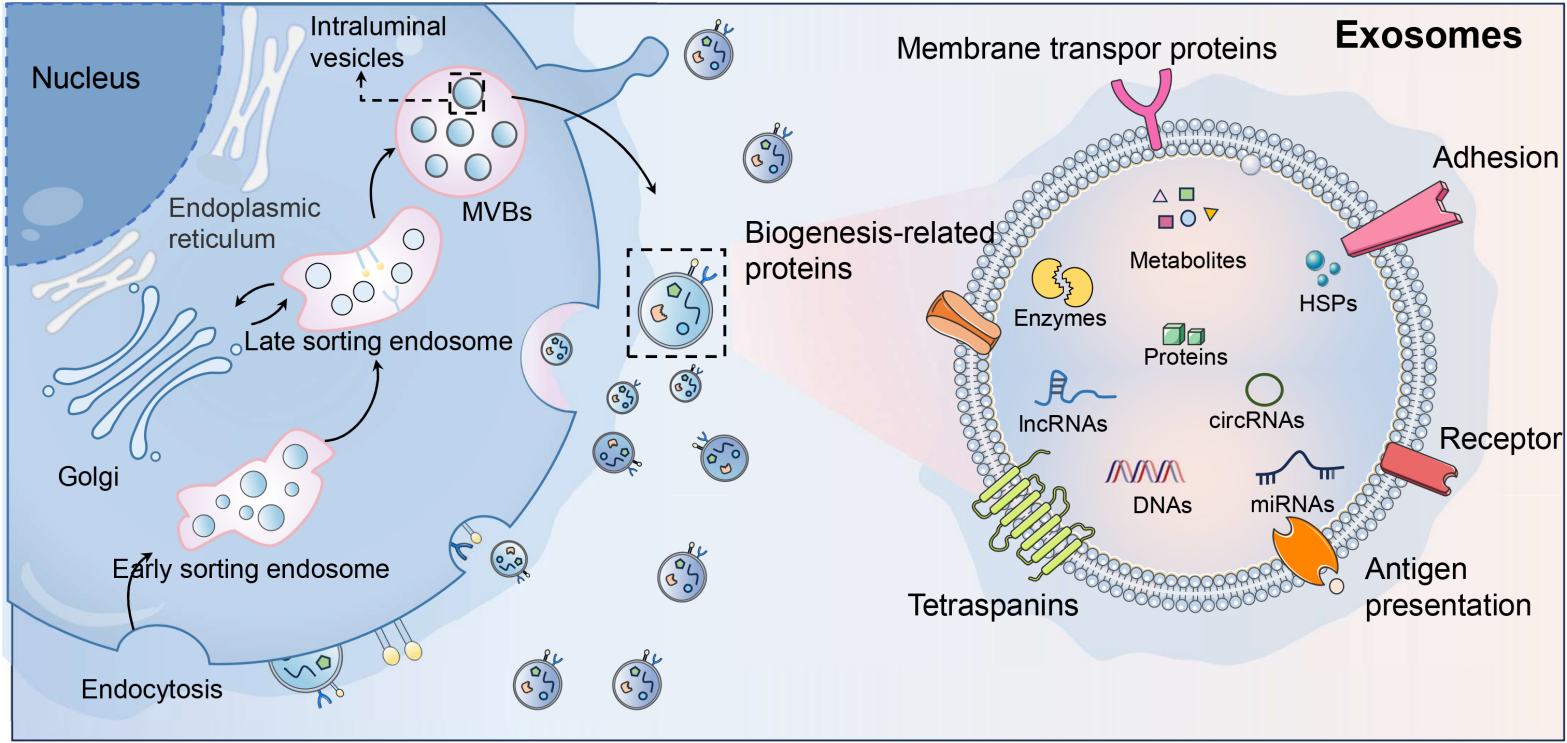
Exosome biogenesis and signature. As the main subtype of extracellular vesicles (EVs), exosomes are key players in intercellular communication within the tumor microenvironment (TME), and originate from the inward budding of the plasma membrane, forming early endosomes that mature into multivesicular bodies (MVBs). The ESCRT machinery and ESCRT-independent pathways involving lipid rafts and tetraspanins (CD63, CD81, CD9) generate intraluminal vesicles (ILVs). MVBs may fuse with lysosomes for degradation or with the plasma membrane for exosome release, regulated by Rab GTPases like Rab27a and Rab27b. Characterized by a cholesterol- and ceramide-rich lipid bilayer, exosomes carry proteins, lipids, and nucleic acids. In the TME, exosomes influence oncogenic pathways, promote angiogenesis, and suppress immune responses, serving as potential therapeutic targets and cancer biomarkers.

In BC, EVs significantly influence TME by facilitating cell-to-cell communication through the transfer of oncogenic signals and regulatory RNAs, thus promoting angiogenesis, immune suppression, and metastasis (25). EVs can polarize macrophages towards a tumor-supportive phenotype and impair immune cells like T cells, weakening the anti-tumor response (26). Their ability to alter recipient cell behavior makes them key players in cancer progression, enhancing tumor growth and facilitating immune evasion. Additionally, EVs hold potential as carriers for therapeutic agents and biomarkers, offering promising avenues for cancer diagnosis and therapy (27). Understanding their features and interactions within the TME provides insights into their role in cancer biology, highlighting them as potential targets for therapeutic intervention. By manipulating exosome productions or cargoes, new cancer treatment strategies can be developed, potentially improving patient outcomes (**Figure 1**).

## 3 Normal mammary epithelia

The disruption and differentiation of normal mammary epithelial cells significantly drive mammary gland development (28). This alteration not only fosters an environment conducive to tumor initiation but also promotes the expansion of progenitor cell populations, facilitating BC progression and metastasis. Bertolini et al. declared that invasive BC cells under hypoxic conditions released small EVs containing hypoxia-inducible factor-1α (HIF-1α), thus then altered normal mammary epithelial differentiation, expanded progenitor cell populations, and induced systemic immunosuppression by increasing S100A9 release from myeloid cells, promoting epithelial-mesenchymal transition (EMT) and luminal cell invasion (29). This process accelerated the onset and progression of bilateral breast cancer, particularly in the presence of the MMTV-PyMT oncogene. Targeting HIF1α in these vesicles or deleting S100A9 restored normal differentiation and immune function, highlighting sEVHIF1α as a potential indicator for luminal BC progression and recurrence risk.

## 4 Osteoprogenitor

Osteoprogenitor cells (OPs) are undifferentiated precursors in the bone marrow with the potential to differentiate into osteoblasts (30). OPs significantly influence BC progression by interacting with tumor cells to modify the bone marrow microenvironment, facilitating bone metastasis and modulating immune cell function through the secretion of specific factors (31). Notably, HTRA1 from remote tumor-released sEVs was delivered to OPs in the bone marrow, and promoted the upregulation of matrix metalloproteinase 13 (MMP-13) in OPs, thus leading to the dislocation of hematopoietic stem cells and aggregation of CD41− granulocyte-monocyte progenitors (GMPs) (32). Consequently, there was an overproduction of immunosuppressive myeloid cells, impairing systemic anti-tumor immunity even after tumor removal. These persistent changes reduced the effectiveness of immunotherapies, necessitating additional interventions to restore immune function. The results showed that inhibiting or conditionally knocking out MMP-13 significantly enhanced immune reinstatement and improved immunotherapy efficacy, emphasizing that tumor-induced comprehensive effects from OP-GMP crosstalk persist beyond tumor presence. Therefore, remote tumor-derived sEVs carrying HTRA1 strongly upregulated MMP-13 in OPs, significantly disrupting the bone marrow ecosystem.

## 5 MDSCs

Myeloid-derived suppressor cells (MDSCs) are a diverse group of immature myeloid cells that expand significantly during tumor progression, facilitating immune evasion and tumor invasion (33). MDSCs are diverse in classification and interact with other myeloid, immune, and stromal cells, forming a central component of the immunosuppressive network (34, 35). Notably, early-stage MDSCs (eMDSCs) are a newly identified subclass of MDSCs with enhanced immunosuppressive capabilities, crucial for early tumor-induced immune evasion by modulating T cell activity and altering the TME. BC-derived exosomes were modified by cytokines such as CC‐chemokine ligand 2 (CCL2), binding to glycosaminoglycan side chains on proteoglycans like CD44 and syndecan-1 (36). Cytokine-decorated exosomes preferentially accumulated in metastatic organs like the lung, and thus targeted CCR2+ immune cells such as MDSCs and natural killer (NK) cells. This engagement fostered a metastasis-supportive environment by reshaping the immune landscape and accelerating metastatic progression. The presence of cytokine-bound exosomes in both cancer patients and healthy individuals suggested a significant role in modulating metastasis-favorable environment. Therefore, exosome-conjugated cytokines were recognized as a key modulator of exosome-cell communications. Furthermore, Jiang et al. also demonstrated that BC cell-derived exosomes could deliver miR-9 and miR-181a to eMDSCs, and trigger the expansion by posttranscriptionally regulating suppressors of cytokine signaling 3 (SOCS3) and protein inhibitor of activated STAT3 respectively (PIAS). These functions activated the JAK/STAT signaling pathway in a negative feedback method, resulting in suppressed T-cell immunity and increased tumor growth and immune escape (37). In 4T1 BC models, miR-9 and miR-181a enhanced eMDSC infiltration and inhibited T-cell immunity *in situ*, thus promoting an immunosuppressive environment. Targeting this exosome-mediated delivery could offer a therapeutic strategy for IL-6^high^ breast cancer.

## 6 Neutrophils

Neutrophils, traditionally seen as simple components of the innate immune system, are now recognized for their complex roles in regulating processes like acute injury, cancer, and chronic inflammation (38). Neutrophils also influence adaptive immunity by shaping specific immune responses, and are linked to poor clinical outcomes, undergoing epigenetic and functional changes to become pro-angiogenic in the TME (39). EVs derived from MDA-MB-231 cells were shown to polarize neutrophils towards a pro-tumor N2 phenotype (40). EVs-impacted neutrophils exhibited increased lifespan, chemotactic ability, and released higher levels of neutrophil extracellular traps (NETs), reactive oxygen species (ROS), IL-8, vascular endothelial growth factor (VEGF), and MMP9, alongside upregulated CD184 expression, ultimately promoting tumor cell migration and viability. Using annexin-V to block phosphatidylserine could mitigate the pro-tumor effects of EVs on neutrophils in BC.

## 7 DCs

Dendritic cells (DCs) are vital antigen-presenting cells that initiate and regulate both innate and adaptive immune responses, playing a crucial role in cancer immunotherapy (41). The TME significantly influences the diverse functions of DC subsets, which can either potentiate antitumor immunity or facilitate immune tolerance and tumor progression (42). EVs are a category of information transporters in impacting DC biology and capabilities (43). Safaei et al. unveiled that exosomes derived from TNBC cells were delivered to monocyte-derived dendritic cells (moDCs), enhancing their maturation and function (44). The exosome-treated moDCs stimulated T-cell expansion by promoting TH1 differentiation through increased cytokine production, suggesting a potential improvement in vaccine-elicited immunotherapy for TNBC. The immunogenic potential of TNBC-derived exosomes on moDCs is pivotal in influencing T-cell responses. Taghikhani et al. utilized electroporation to induce the overexpression of miR-155, miR-142, and let-7i in tumor-derived exosomes from 4T1 cells, enhancing their ability to promote DC maturation (45). As a result, these modified exosomes effectively facilitated tumor immune cytotoxicity.

**Figure 2:**
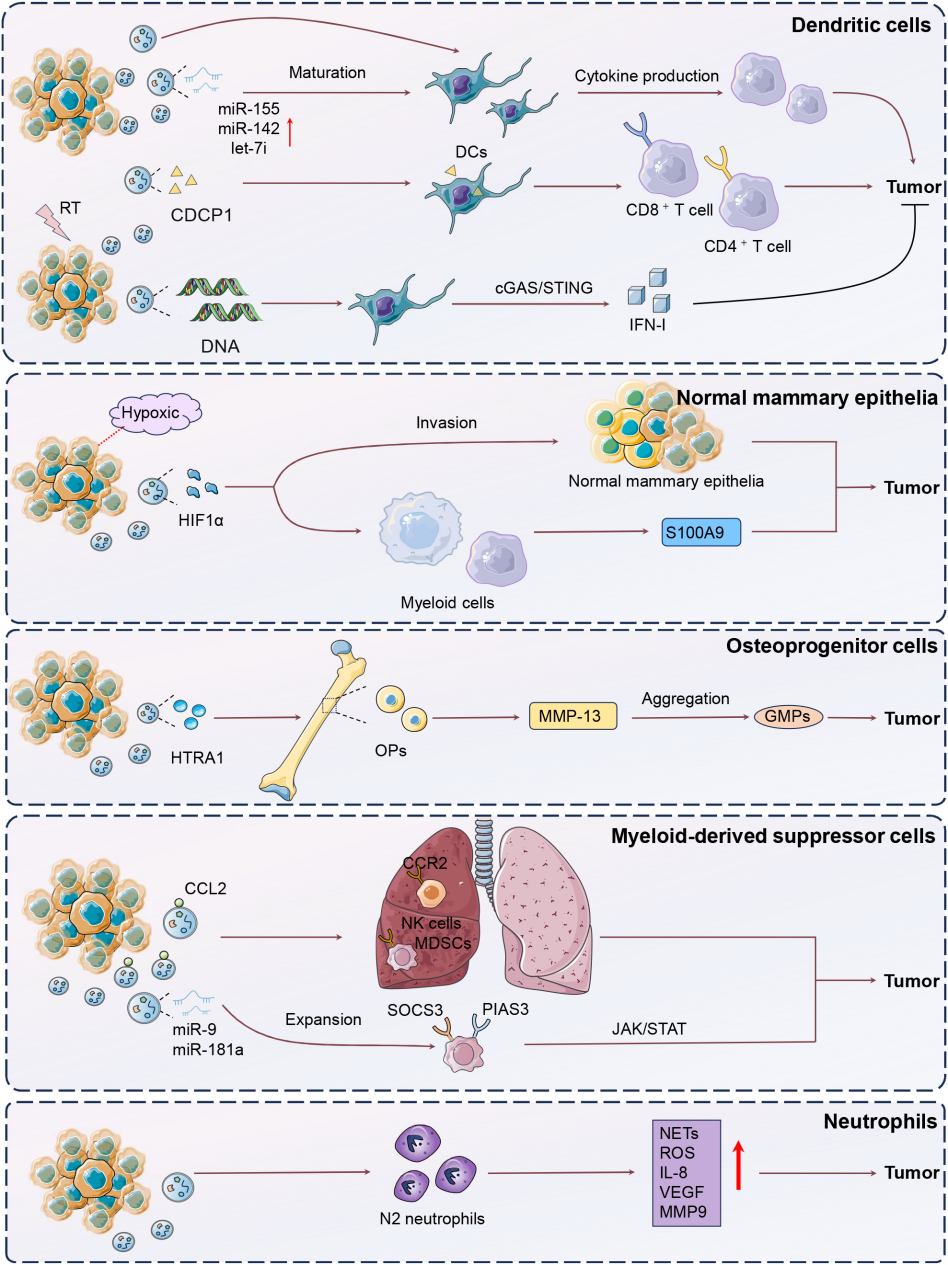
Mechanisms of DCs, neutrophils, MDSCs, osteoprogenitor cells modulation by tumor-derived EVs. The figure illustrates the complex interactions between breast cancer (BC) cells and various immune components through extracellular vesicles (EVs), highlighting their roles in tumor progression and immune modulation. In BC, hypoxic conditions lead to the release of EVs containing hypoxia-inducible factor-1α (HIF-1α), which disrupts normal mammary epithelial differentiation, expands progenitor cell populations, and induces systemic immunosuppression by promoting S100A9 release, epithelial-mesenchymal transition (EMT), and luminal cell invasion. Osteoprogenitor cells (OPs) in the bone marrow, influenced by tumor-derived EVs containing HTRA1, upregulate matrix metalloproteinase 13 (MMP-13), leading to immunosuppressive myeloid cell production and impaired immunotherapy efficacy. Myeloid-derived suppressor cells (MDSCs), expanded by BC-derived exosomal miRNAs such as miR-9 and miR-181a, suppress T-cell immunity through the JAK/STAT pathway, contributing to immune evasion. Neutrophils are polarized by EVs to a pro-tumor N2 phenotype, enhancing tumor viability through increased NETs, ROS, and VEGF production. Dendritic cells (DCs), influenced by exosomes from triple-negative breast cancer (TNBC) cells, enhance T-cell responses and promote antitumor immunity. Irradiated tumor cells release EVs that deliver tumor antigens and heat-shock proteins to DCs, increasing T-cell infiltration and inducing tumor-specific immunity, with CDCP1 identified as a novel tumor-associated antigen.

In addition, Irradiated tumor cells released sEVs that enhanced antitumor immunity by delivering tumor antigens and heat-shock proteins (46). Transferred sEVs were internalized by DCs, and then increased CD8+ and CD4+ T cell infiltration, effectively targeting primary tumors and lung metastases. Notably, CUB-domain-containing protein 1 (CDCP1) from radiation-induced sEVs was identified as a novel tumor-associated antigen, contributing to their capability of inducing tumor immunogenicity. Similarly, irradiated murine BC cells produced tumor exosomes (RT-TEX) that transferred double-stranded DNA to DCs, leading to the upregulation of co-stimulatory molecules and STING-dependent activation of type I interferons (47). *In vivo*, RT-TEX elicited a tumor-specific CD8+ T cell response, offering superior protection against tumor development in vaccination experiments compared to exosomes from untreated cancer cells. These findings reveal RT-TEX as a pathway for delivering IFN-stimulatory DNA from irradiated tumor cells to DCs, enhancing anti-tumor immunity (**Figure 2**).

## 8 Macrophages

### 8.1 NcRNAs in tumor-derived EVs

BC progression is intricately influenced by the TME, where EV-derived miRNAs are critical in modulating cellular behaviors, such as proliferation, invasion, and drug resistance (48). Non-coding RNAs (ncRNAs), including micro-RNAs (miRNAs) and long noncoding RNAs (lncRNAs), are key regulators of both intracellular and intercellular signaling, affecting processes like estrogen receptor activity and tumor cell communication (49). Understanding the functions and therapeutic potential of EV-derived ncRNAs could lead to innovative strategies for BC diagnosis and treatment.

Tumor-derived EV-miRNAs are small non-coding RNA molecules, and function in post-transcriptional gene regulation by binding to target mRNAs, thus shaping the TME by influencing immune cell behavior and facilitating cancer progression. Xing et al. established that knockout of X-inactive-specific transcript (XIST) accelerated primary tumor growth and brain metastasis, accompanied by EMT and activated c-Met via MSN-mediated protein stabilization, leading to increased tumor stemness (50). Additionally, exosomal miRNA-503 secretion was elevated following XIST knockout, inducing M1-M2 polarization of microglia and upregulating immunosuppressive cytokines, thereby inhibiting T cell proliferation. Thus, exosomal miRNA-503 and XIST were critical regulators of the TME and BC brain metastasis. Exosomes from BC cells experiencing endoplasmic reticulum stress delivered miR-27a-3p to macrophages, leading to increased PD-L1 expression (51). Then, T cell activity was inhibited by targeting the MAGI2/PTEN/PI3K pathway, facilitating immune escape. Consequently, this mechanism promoted tumor growth by diminishing effective immune surveillance. Exosomes derived from EGCG-treated 4T1 BC cells transferred miR-16 to tumor-associated macrophages (TAMs), reducing their infiltration and M2 polarization (52). Then, there were decreased IL-6 and TGF-β levels and increased TNF-α expression, shifting TAMs towards an M1-like phenotype. Resultantly, exosomes derived from EGCG-treated 4T1 BC cells could restrain tumor development by curbing TAM infiltration and M2 polarization.

Additionally, exosomes derived from 4T1 BC cells delivered miR-33 mimics to IL-4-induced M2 macrophages, effectively converting them to the M1 phenotype (53). This conversion was evidenced by increased expression of M1 markers (Irf5, Nos2, CD86) and cytokines (TNF-α, IL-1β), alongside reduced M2 markers (Arg, Ym1, CD206) and cytokines (IL-10, TGF-β). Consequently, the conditioned media from these repolarized macrophages inhibited the proliferation, invasion, and migration of 4T1 BC cells, highlighting the potential of exosome-mediated miR-33 delivery in antitumor strategies. Similarly, Tumor-derived exosome-delivered miR-130 to M2 macrophages, also resulting in their repolarization to the M1 phenotype, along with enhanced phagocytic activity and reduced the migration and invasion capabilities of BC cells (54).

Circular RNAs (circRNAs), a subclass of covalently closed, endogenous noncoding RNAs capable of microRNA sponging, are abundant in tumor-derived EVs (55). CircRNAs in tumor-derived EVs are important modulators in gene regulation, impacting carcinogenesis, metastasis, and treatment resistance (56). Exosomal circRNAs enhance cell communication and tumor spread in BC (57). Zhuang et al. showed that exosomal circ-0100519, originating from BC cells, significantly promoted tumor progression by inducing M2 macrophage polarization through the USP7/NRF2 axis (58). This circRNA acted as a scaffold, enhancing the interaction between USP7 and NRF2, which led to NRF2 deubiquitination and increased BC cell invasion and metastasis. Additionally, HIF-1α was identified as an upstream regulator that enhanced circ-0100519 transcription.

### 8.2 Proteins in tumor-derived EVs

M2 macrophage polarization and enhanced IL-6 expression drive tumor progression, highlighting their crucial role in shaping the TME across various cancers (59, 60). BC-derived exosomes transformed macrophage phenotypes by activating the IL-6/STAT3 pathway (61). These exosomes were highly enriched with gp130, induced bone marrow-derived macrophages (BMDMs) to secrete IL-6 and enhanced their survival. Adding a gp130 inhibitor to the cancer-derived exosomes or blocking BMDM uptake could reverse these effects. The study confirmed that breast cancer-derived exosomes mediated macrophage polarization via the gp130/STAT3 pathway. Tumor-derived exosomes were internalized by macrophages in axillary lymph nodes, inducing IL-6 expression (62). The deletion of toll-like receptor 2 (TLR2) or MyD88 completely abolished this effect, while palmitoylated proteins on the exosome surface facilitated NF-κB activation. Therefore, BC cell-derived exosomes activated macrophage immune responses through TLR2-mediated NF-κB pathways.

Recent advances in tumor immunotherapy highlight the limited efficacy of PD-1/PD-L1 inhibitors in solid cancers, largely due to the influence of TAMs within TME (63). Exosome-mediated M2 macrophage polarization is involved in this phenomenon (64). Morrissey et al. pointed out that tumor-derived exosomes reprogrammed tissue-resident macrophages within the pre-metastatic niche to an immunosuppressive phenotype by enhancing glycolysis through NF-kB signaling (65). This reprogramming led to increased PD-L1 expression, elevated glucose uptake, and enhanced conversion of pyruvate to lactate, which further boosted PD-L1 levels. These results highlight a mechanism by which TDEs promote a pro-metastatic environment, linking exosomal signaling to metabolic changes and immune modulation.

Chemokines are crucial in tumor metastasis by modulating TAMs and other immune cells, fostering an immunosuppressive TME that enhances cancer spread and identifies specific targets for checkpoint blockade therapies. In patient-derived xenograft models of TNBC with varying metastatic propensities, TNBC-derived EVs induced TNFα expression in the liver with upregulated CX3CL1 expression (66). This cascade facilitated the recruitment of CX3CR1-expressing macrophages, enhancing MMP9 expression and promoting macrophage migration and cancer cell invasion. TNBC-derived EVs resulted in premetastatic niche formation in the liver via TNF-α-induced CX3CL1-CX3CR1, highlighting their potential as therapeutic targets in preventing hepatic metastasis. In addition, Rabe et al. showed that tumor-derived EVs programmed naïve macrophages into a pro-metastatic phenotype in triple-negative breast cancer (TNBC) by modulating CCL5 expression (67). These EV-educated macrophages secreted factors like CXCL1 and TGFβ, which remodeled the tumor stroma and immune environment and enhanced tumor invasion. Consequently, injecting these educated macrophages into mice significantly increased lung metastasis. Tkach et al. showed that EVs from TNBC cells were able to induce pro-inflammatory macrophages by delivering surface colony stimulating factor-1 (CSF-1) and activating cargoes to monocytes (68). EV-induced macrophages exhibited an interferon response signature, enhancing T cell infiltration within the TME. Furthermore, macrophages expressing the EV-induced signature were present in TAMs from patients and correlated with improved survival outcomes. Thus, TNBC-derived EVs with CSF-1 expression contributed to a more favorable immune response, and determined the differentiation fate of macrophages. Thus, chemokines such as CX3CL1, CCL5, and CXCL1 are involved in the actions of EVs-mediated premetastatic niche formation, enhanced tumor invasion, and modulated immune responses.

### 8.3 Drug resistance mediated by tumor-derived exosomes

Exosomes significantly influence drug resistance by delivering proteins, RNAs, and lipids that modulate immune cell activity and alter drug metabolism pathways (69). These vesicles can carry efflux transporters and anti-apoptotic factors, directly impacting therapeutic efficacy and enhancing cancer cell survival against drug treatments (70). Inhibiting the biogenesis or signaling of tumor-derived EVs can reverse drug resistance, including that seen with chemotherapeutics like cisplatin, paclitaxel, and doxorubicin.

After treatment with paclitaxel or adriamycin, apoptotic TNBC cells released EVs (EV-dead) containing CXCL1, and were phagocytized by macrophages, leading to increased infiltration of immunosuppressive PD-L1+ TAMs, which promoted TNBC metastasis (71). Lastly, targeting CXCL1 in chemotherapy-elicited EVs with TPCA-1 could inhibit TAM/PD-L1 signaling, thus enhancing chemosensitivity and reducing TNBC metastasis. They further confirmed that paclitaxel treatment led to the release of CXCL1-enriched EVs from apoptotic TNBC cells (EV-Apo), which were taken up by macrophages, promoting their polarization to the M2 phenotype and enhancing tumor chemoresistance and invasion (72). Baohuoside I (BHS) effectively reduced CXCL1 levels in these vesicles, thereby inhibiting PD-L1 activation and M2 macrophage polarization. Additionally, BHS disrupted the biogenesis of intraluminal vesicles, diminishing overall EV release, and BHS-mediated disruption significantly increased paclitaxel chemosensitivity and inhibited TNBC metastasis, highlighting BHS as a potential adjuvant for improving therapeutic outcomes via inhibiting EV-Apo^CXCL1^ biogenesis via directly interfering with the RAB31/FLOT2 interaction. XIAOPI formula (XPS) effectively reduced CXCL1 levels in EV-dead released from apoptotic TNBC cells, diminishing their ability to induce M2 macrophage polarization and PD-L1 expression (73). Consequently, XPS significantly enhanced paclitaxel efficacy, inhibiting TNBC growth and metastasis *in vivo*, mainly by suppressing EV-dead^CXCL1^-induced PD-L1 activation and M2 polarization of macrophages.

**Figure 3:**
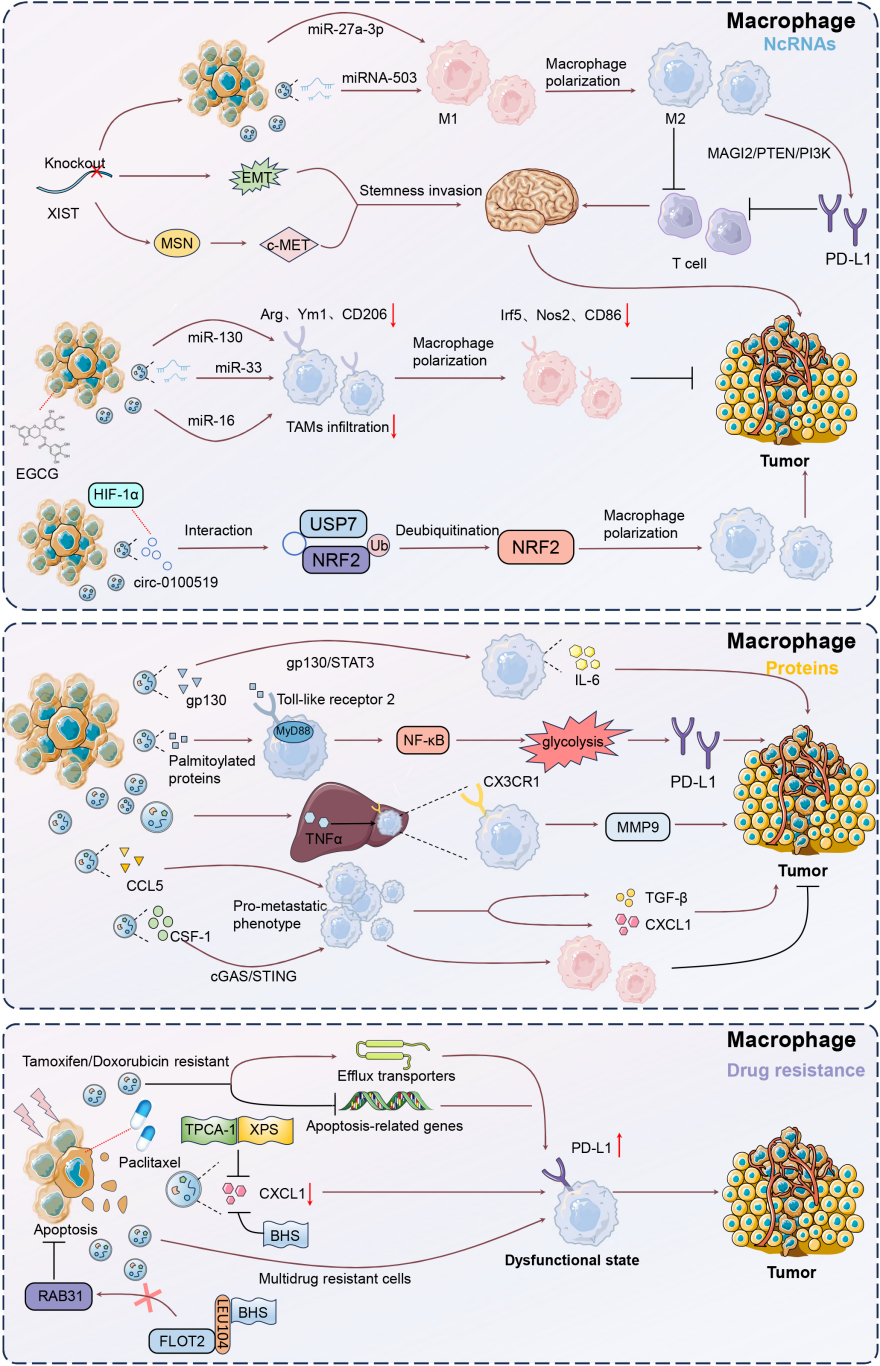
Mechanisms of macrophage modulation by tumor-derived EVs. Upon uptake by macrophages, EVs can induce a shift towards a tumor-supportive M2 phenotype, characterized by increased secretion of immunosuppressive cytokines such as IL-6 and TGF-β. This polarization is mediated through pathways like gp130/STAT3 and NF-κB, enhancing tumor growth and immune evasion. Additionally, EVs can deliver microRNAs, such as miR-130 and miR-33, which further promote M1 to M2 transition, impacting anti-tumor immunity. Understanding these interactions offers potential therapeutic targets for reprogramming macrophages to support anti-tumor responses, thereby improving cancer treatment outcomes.

Microparticles (MPs) released from both cancerous and non-cancerous cells triggered the secretion of pro-inflammatory cytokines upon transfer to macrophages (74). Especially, MPs from multidrug-resistant (MDR) tumor cells specifically drove macrophages toward a dysfunctional state, enhancing their phagocytosis by foreign cells. Santos et al. posed that EVs derived from tamoxifen- and doxorubicin-resistant BC cells enhanced drug resistance in sensitive MCF-7 and MDA-MB-231 cells by upregulating efflux transporters and downregulating apoptosis-related genes (75). These EVs also altered immune signaling, facilitating immune evasion and promoting cell survival. Furthermore, they induced a pro-inflammatory cytokine response in macrophages, suggesting a potential target for developing targeted immunotherapies (**Figure 3**).

## 9 T cells

### 9.1 NcRNAs in tumor-derived EVs

Clinically, high Lin28B and low let-7s levels correlated with poor prognosis and increased lung metastasis risk in BC patients (76). BC cells with high Lin28B expression released exosomes with low let-7s levels, promoting immune suppression in the lung pre-metastatic niche (76). These exosomes facilitated the recruitment and conversion of neutrophils to an N2 phenotype, which upregulated PD-L2 and altered the cytokine environment, further forming immune suppression to support lung metastasis by creating a favorable niche for cancer progression. Ni et al. also showed that exosomes from BC cells could deliver lncRNA SNHG16 to Vδ1 T cells, inducing their transformation into CD73+ immunosuppressive cells (77). This transformation was mediated through the activation of the TGF-β1/SMAD5 pathway, facilitated by SNHG16 sponging of miR-16–5p. As a result, the CD73+γδ1 T cells enhanced the immunosuppressive microenvironment by producing adenosine. These findings suggested that disrupting exosomal signaling or targeting the CD73+γδ1 T cell population could benefit tumor treatment strategies.

### 9.2 Proteins in tumor-derived EVs

In BC TME, CD8+ T cells can internalize proteins from tumor-derived EVs, leading to alterations in their function (78). These interactions often result in metabolic and signaling changes that contribute to immune evasion and tumor progression (79). Choudhury et al. showed that BC cell-derived exosomes could transfer functional molecules to activated CD8+ T cells, leading to a reduction in their glycolytic activity through the downregulation of the AKT/mTOR pathway (80). The metabolic reprogramming impaired the effector functions of CD8+ T cells, contributing to immune evasion and facilitating tumor progression. Thus, inhibiting BC-derived EVs improves tumor control with enhanced CD8+ T cell responses.

Malignant BC cells secreted EVs carrying active TGF-β type II receptors, which were absorbed by low-grade tumor cells, triggering EMT and enhancing metastasis (81). These vesicles also transferred TβRII to CD8+ T cells, activating SMAD3 and TCF1, leading to T cell exhaustion and reduced immunotherapy efficacy. The presence of TβRII+ EVs correlated with increased tumor burden and metastasis, highlighting a novel mechanism for T cell exhaustion and dampened anti-tumor immunity. Najaflou et al. confirmed that in 4T1 xenograft mouse models, exosomes derived from photothermal-treated 4T1 BC cells exhibited enhanced levels of damage-associated molecular patterns (DAMPs), including HSP70, HSP90, and high-mobility group box 1 (HMGB-1) (82). These exosomes significantly increased T-cell infiltration and cytokine expression IL-6, IL-12, and IL-1β within tumors, leading to inhibited tumor progression. Besides, photothermal-derived exosomes are more effective than hyperthermia-derived exosomes in stimulating immune responses and suppressing tumor progression.

TNBC cells released exosomes enriched with intercellular adhesion molecule 1 (ICAM1) that induced CD8+ T cell exhaustion and suppressed their proliferation and activation (83). This interaction fostered an immunosuppressive microenvironment, facilitating tumor growth and the spread of cancer to the bones. Blocking ICAM1 significantly reduced the exosome capability to inhibit CD8+ T cells, highlighting a potential target for immunotherapy to prevent TNBC bone metastasis. Exosomes isolated from BC cells were found to contain ectonucleotide pyrophosphatase phosphodiesterase 1 (ENPP1), which hydrolyzed both synthetic and endogenous cGAMP, thereby inhibiting the cGAS-STING signaling pathway in immune cells (84). This hydrolytic activity extended to cGAMP bound to LL-37, further suppressing STING activation and reducing the infiltration of CD8+ and CD4+ T cells. The high expression of ENPP1 in these exosomes facilitated immune evasion, potentially impacting tumor growth and metastasis. Consequently, tumor-derived exosomal ENPP1 modulates immune responses by hydrolyzing cGAMP to inhibit cGAS-STING signaling. Xie et al. proved that ubiquitin-specific peptidase 8 (USP8) enhanced BC metastasis by deubiquitinating and stabilizing the TGF-β receptor TβRII, thus leading to increased TGF-β/SMAD signaling (85). USP8 facilitated the secretion of TβRII+ EVs, which induced CD8+ T cell exhaustion and contributed to chemoimmunotherapy resistance. Pharmacological inhibition of USP8 reduced TβRII stability, decreased TGF-β signaling, and alleviated T cell exhaustion. Consequently, targeting USP8 improved the efficacy of BC immunotherapy and suppressed tumor progression. In this study of Raiter et al., EVs derived from MDA-MB-231 TNBC cells and patient plasma were found to contain Galectin 3 binding protein (Gal3BP), which could interact with Galectin 3 (Gal3) (86).

Regulatory T (Treg) cells, characterized by markers such as CD25, FOXP3, and CTLA-4, are crucial inhibitory cells that maintain self-tolerance and modulate adaptive immunity (87). EVs-carried Gal3BP/Gal3 complex induced immunosuppression by increasing Treg and suppressive IL-10 and IL-35 in peripheral blood mononuclear cells (PBMCs). Blocking the CD45 receptor on PBMCs exposed to tumor-derived EVs reversed this immunosuppression, enhancing IFN-γ production and activating CD4, CD8, and CD56 effector cells. This study revealed a potential tumor escape mechanism involved in the interaction between CD45 receptor and Gal3BP/Gal3 complex, specifically in peripheral leukocytes.

### 9.3 PD-L1 in tumor-derived EVs

PD-L1 is a protein expressed on the surface of various cells, including tumor cells, that binds to the PD-1 receptor on T cells (88). This interaction inhibits T cell activity, allowing cancer cells to evade the immune response and promoting tumor growth. PD-1/PD-L1 antibodies have revolutionized cancer therapy by reactivating T cells and enhancing antitumor immunity, yet their efficacy varies due to cancer complex immune evasion strategies (89). PD-L1 in Tumor-derived EVs impacts immune evasion by targeting PD-1 on CD8+ T cells, leading to immunosuppression and resistance to PD-1/PD-L1 checkpoint therapies (90).

Exosomes derived from BC cells were found to transfer PD-L1 to other cells, including those with low or no PD-L1 expression, such as MCF-7 and BT549-PD-L1KO cells (91). This transfer led to the suppression of T cell activation and cytotoxicity, thereby promoting tumor growth by reducing granzyme B secretion from T cells. Notably, BC cells released exosomes rich in PD-L1 under the influence of transforming growth factor beta (TGF-β), contributing to CD8+CD39+ and CD8+PD1+ T-cell dysfunction (92). Exosomal PD-L1 was linked to higher TGF-β levels within TME. Blocking TGF-β and exosome release *in vivo* reduced tumor burden by boosting Granzyme and IFN-γ production. Therefore, TGF-β enhanced PD-L1 in tumor exosomes and disrupted CD8 T-cell function by affecting early T cell receptor (TCR) signal phosphorylation in breast cancer. Zhao et al. reported that EVs from TNBC cells significantly increased PD-L1 expression, causing a notable reduction in CD8+ T cell cytotoxicity and promoting immune evasion (93). Upregulation of VAMP3 restored paclitaxel (PTX) resistance, likely due to autophagy activation, as the autophagy inhibitor chloroquine enhanced PTX sensitivity. CCAAT enhancer binding protein delta (CEBPD) was found to bind to the VAMP3 promoter, activating its transcription, and this CEBPD/VAMP3 axis also elevated PD-L1 levels in the conditioned media of TNBC cells. Thus, CEBPD/VAMP3 axis was a crucial pawthay to mitigate immune suppression and enhance therapeutic efficacy in TNBC. These findings confirmed the role of exosomal PD-L1 in modulating immune responses and highlighted its potential impact on tumor progression and resistance to immunotherapy.

Microvesicles or microparticles, derived from the cytoplasmic membrane of cancer or normal cells, carry diverse biomolecules and are frequently released in TME, significantly impacting tumor biology and immunology (94). Following radiotherapy, tumor-derived MPs from breast carcinoma cells carry elevated levels of immune-modulating proteins, including PD-L1, and are delivered to cytotoxic T lymphocytes (CTLs) (95). This interaction inhibited CTL activity, leading to enhanced tumor growth both *in vitro* and *in vivo*. Importantly, blocking the PD-1/PD-L1 axis partially reversed this inhibition, highlighting a potential therapeutic synergy between radiotherapy and immune checkpoint inhibitors to counteract tumor immune evasion. TNBC cell-released MPs carrying the immune checkpoint molecule PD-L1, particularly following chemotherapy and radiotherapy, suppressed CD8+ T cell activation and function, and induced macrophage differentiation into the immune-suppressive M2 phenotype via the TBK1/STAT6 and AKT/mTOR pathways (96). This immune suppression facilitated tumor progression, but combining chemotherapy with the PD-L1 inhibitor atezolizumab effectively countered this effect. Collectively, this study deciphered a novel immune surveillance mediated by PD-L1-loading MPs in TNBC immunotherapy.

**Figure 4:**
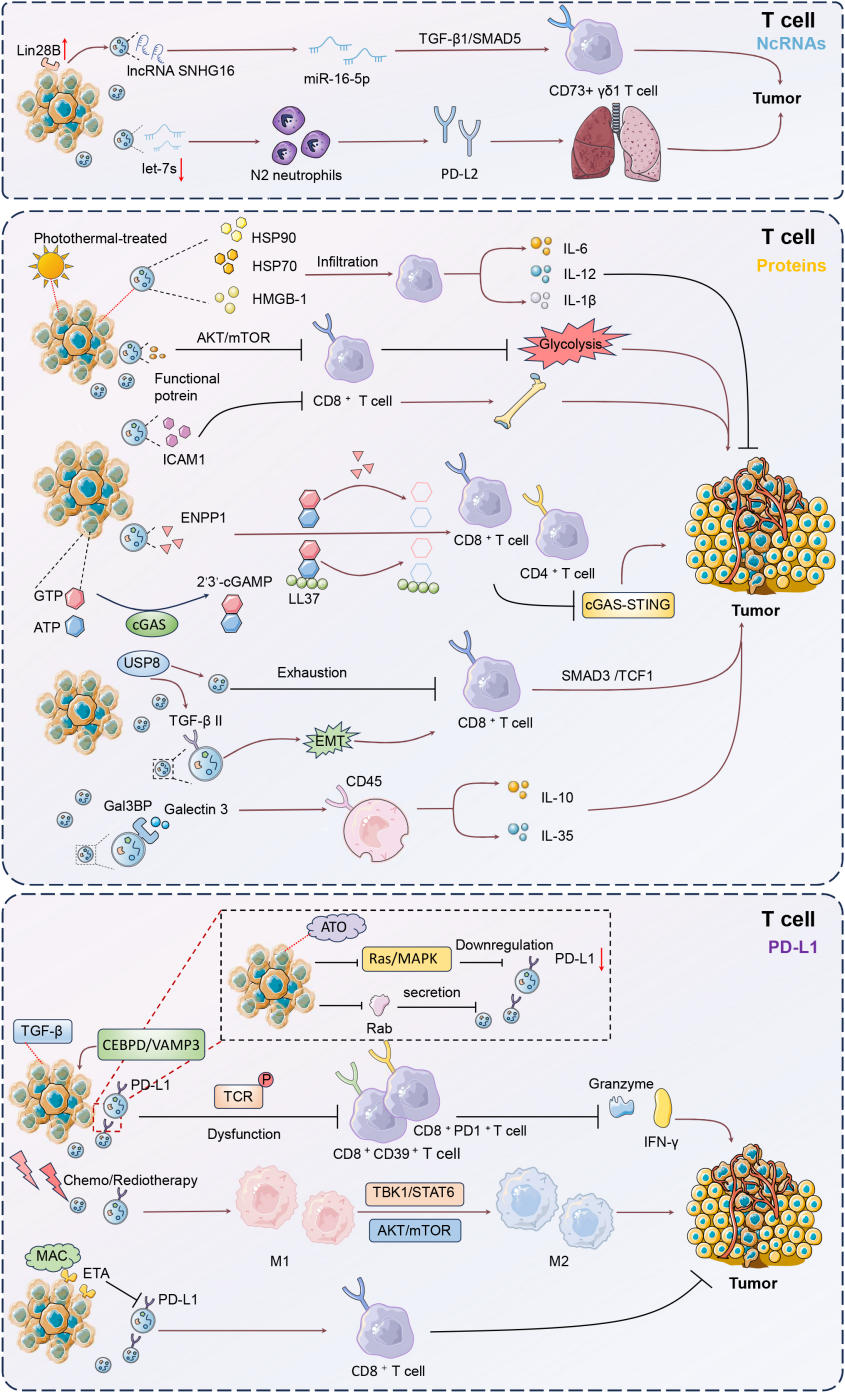
Mechanisms of T cell modulation by tumor-derived EVs. Tumor-derived EVs critically influence T cell function within the tumor microenvironment (TME). These EVs transport non-coding RNAs and proteins that modulate T cell activity, promoting immune evasion and tumor progression. EVs carrying lncRNA SNHG16 can transform Vδ1 T cells into CD73+ immunosuppressive cells via the TGF-β1/SMAD5 pathway, enhancing an immunosuppressive milieu. Additionally, EVs containing TGF-β type II receptors induce CD8+ T cell exhaustion through SMAD3 activation, undermining immunotherapy efficacy. PD-L1-rich EVs further suppress T cell cytotoxicity, facilitating tumor growth and metastasis. Targeting these pathways, such as inhibiting the CEBPD/VAMP3 axis or blocking PD-L1 interactions, offers potential therapeutic strategies to restore T cell function and improve treatment outcomes in breast cancer.

Under the influence of specific therapeutic interventions, tumor-derived exosomes exhibit notable structural and functional transformations, including a marked reduction in PD-L1 expression. This modulation diminishes the exosome-mediated immunosuppressive capabilities, and potentiates the antitumor immune responses. For instance, Choe et al. declared that BC cell-derived EVs exhibited reduced PD-L1 levels and secretion when treated with atorvastatin, and enhanced the efficacy of immune checkpoint therapy (97). This effect was probably achieved by regulating Rab proteins involved in EV biogenesis and inhibiting the Ras-activated MAPK signaling pathway to downregulate PD-L1 expression. As a result, ATO improved antitumor immunity by boosting T-cell activity, particularly enhancing the response to anti-PD-L1 therapy. Lee et al. also showed that EVs derived from tumor cells carried PD-L1, which interacted with PD-1 on CD8+ T cells, significantly reducing their antitumor activity (98). Macitentan inhibited the secretion of EV PD-L1 by targeting endothelin receptor A (ETA), thereby enhancing CD8+ T cell-mediated tumor killing and improving the efficacy of PD-L1 blockade in BC models (98). This resulted in increased CD8+ T cell numbers and decreased regulatory T cell presence, leading to significant tumor reduction in TNBC, colon, and lung cancer models (**Figure 4**).

## 10 Limitations and perspectives

This study delves into the intricate and sophisticated roles of tumor-derived EVs in reshaping immune modulation and driving BC progression, through several key mechanisms. Firstly, EVs facilitate immune modulation by transporting molecules like PD-L1 and miRNAs such as miR-9 and miR-181a, which suppress T-cell activity and promote the expansion of MDSCs, thereby enhancing immune evasion. Additionally, EVs induce macrophage polarization towards a tumor-supportive M2 phenotype, further weakening the anti-tumor immune response. In terms of angiogenesis, EVs could deliver pro-angiogenic factors like VEGF and IL-8 to endothelial cells, increasing vascularization and supplying tumors with essential nutrients and oxygen for rapid growth. For metastasis and colonization, EVs alter recipient cell behavior and prepare distant sites for tumor colonization by carrying molecules such as TGF-β receptors and ICAM1, inducing EMT and enhancing metastatic potential. They also modify the pre-metastatic niche by delivering chemokines like CX3CL1, which recruit immune cells that support tumor invasion and spread. EVs also contribute to metabolic reprogramming by delivering metabolic enzymes and regulatory RNAs, altering the metabolic landscape of the TME to support cancer cell proliferation and survival under nutrient-deprived conditions. Lastly, EVs play a role in drug resistance by transporting proteins and RNAs that modulate drug metabolism pathways, carrying efflux transporters and anti-apoptotic factors that enhance cancer cell survival against chemotherapy. In summary, tumor-derived EVs are dynamic entities that modulate the TME through immune suppression, promotion of angiogenesis, enhancement of metastatic potential, and contribution to drug resistance. Understanding these EV-mediated pathways offers new avenues for therapeutic intervention, potentially improving patient outcomes by effectively targeting these processes. However, some issues governing EV biogenesis, function, targeted therapy, and clinical potential, should also be addressed.

Firstly, understanding the intricate biogenesis and release mechanisms of EVs is paramount for the development of sophisticated therapeutic interventions. By precisely targeting these processes, we can potentially attenuate the secretion of tumor-derived EVs, thereby diminishing their contributory role in oncogenesis and metastasis. Despite significant advancements, the molecular constituents of EVs, particularly those involved in immune modulation, remain largely enigmatic. For instance, In osteosarcoma and breast carcinoma cells with high IRF5 expression, tumor-derived EVs are less secreted and altered in composition, reducing their pro-metastatic potential (99). These vesicles influence the pre-metastatic microenvironment by suppressing immune evasion mechanisms and decreasing metastatic colonization, particularly in the lungs, highlighting IRF5 critical role in reducing tumor metastasis and improving patient survival. Employing cutting-edge sequencing technologies and proteomic analyses is imperative to elucidate these components.

Especially, in highly heterogeneous subtype TNBC, tumor-derived EVs play a crucial role in tumor progression and immunomodulation. These EVs are enriched with molecules such as PD-L1 and TGF-β, which inhibit T cell activity and promote immune evasion. Additionally, they transport specific long non-coding RNAs and proteins like SNHG16 and ICAM1, altering immune cell functions to enhance immunosuppression. TNBC-derived EVs also induce macrophage polarization towards an M2 phenotype, creating a tumor-supportive environment via IL-6/STAT3 and NF-κB pathways. Moreover, they facilitate metastasis by carrying molecules like CX3CL1, which prepare distant organs for tumor invasion through chemokine and cytokine network modulation. These EVs contribute to drug resistance by transporting molecules such as CXCL1, affecting treatment efficacy and altering the TME to enhance chemoresistance. Understanding these mechanisms highlights the multifaceted roles of EVs in TNBC and reveals potential therapeutic targets.

Then, EVs present a dichotomous influence within BC biology, facilitating both tumor proliferation and metastatic dissemination, while paradoxically exerting tumor-suppressive effects under certain conditions. This multifaceted behavior corroborates the intricate and context-dependent nature of EV functions. A comprehensive elucidation of the molecular and environmental determinants that govern these divergent roles is essential. The specific pathways and contextual variables that modulate EV activity, can unlock their full therapeutic potential. For example, TNBC exosomal circPSMA1 facilitated tumorigenesis, metastasis, and migration by modulating the miR-637/Akt1/β-catenin axis (100). In BC cells, activation of mGluR3 triggered the Rab27-dependent release of EVs containing mitochondrial DNA (mtDNA), facilitated by PINK1 (101). These EVs transferred invasive properties to recipient tumor cells by activating TLR-9, enhancing endosomal trafficking of pro-invasive receptors. This mechanism pointed out that EV-mediated altered metabolism in cancer cells could propagate invasiveness, contributing to the progression of mammary carcinoma. The presence of continuous-digging RNA and proteins by sequencing, represented by circPSMA1 and mtDNA, disrupted immune regulation, promoting an immunosuppressive microenvironment and correlating with poor prognosis in TNBC patients.

In BC, tumor-derived EVs are primarily studied for their role in immune modulation, but their potential impact on stromal cells warrants exploration. Stromal cells support and promote tumor growth within the TME. Although direct reports are scarce, it is hypothesized that EVs may influence stromal cell behaviors, such as proliferation, migration, and paracrine signaling, thereby indirectly shaping the immune milieu. For instance, EVs might stimulate cancer-associated fibroblasts (CAFs) to release pro-inflammatory cytokines, altering local immune cell activity. Moreover, EVs might interact with endothelial cells to affect tumor angiogenesis, subsequently influencing immune cell infiltration and distribution. By modulating endothelial cell function, EVs may alter vascular permeability and homeostasis, affecting the migration and activity of immune cells like T cells and macrophages within tumors. These hypothetical mechanisms suggest a complex network of EV-mediated interactions between stromal and immune cells, despite the lack of direct experimental evidence. Additionally, EVs may interact with mesenchymal stem cells (MSCs), impacting immune regulation within the TME. MSCs, often reprogrammed in tumors, may secrete immunosuppressive factors such as IL-10 and TGF-β, with EVs potentially enhancing this effect by delivering specific miRNAs or proteins that boost MSC immunosuppressive functions, thus facilitating tumor immune evasion (102). Furthermore, EVs might influence the metabolic activity of stromal cells, indirectly affecting immune cell function. By altering the metabolic state of stromal cells, EVs could impact local nutrient and oxygen availability, which may modulate immune cell activity and function (103). For instance, EVs might enhance glycolytic activity in stromal cells, leading to acidification of the TME, thereby inhibiting effector T cell function. These potential mechanisms highlight the complexity of EV-mediated indirect immune modulation in breast cancer, providing directions for future research despite current gaps in direct evidence.

In addition to the well-documented influence of tumor-derived EVs on immune modulation, EVs originating from immune cells themselves can profoundly affect tumor dynamics. Exosomes originating from DCs, macrophages, and T cells have been reported to modulate BC metabolism, drug resistance, and immune responses through various pathways, thus influencing tumor progression. This bidirectional interaction reveals the intricate complexity of TME, necessitating a comprehensive approach that considers EVs from both origins in therapeutic development. Exploring the mechanisms through which immune cell-derived EVs impact tumor cells may reveal novel therapeutic targets, shedding light on the intricate interplay between the immune system and cancer.

The clinical implications of EVs in the realms of diagnosis and therapy are extensive and promising. EVs hold the potential to significantly enhance the efficacy of immune checkpoint inhibitors by modulating the expression and function of checkpoint molecules on immune cells. This synergistic interaction could lead to substantial improvements in immunotherapy outcomes, offering a powerful strategy to overcome existing therapeutic resistance. Further exploration in this area could catalyze breakthroughs in cancer treatment, providing novel strategies to improve patient prognosis and expand the arsenal of tools available to clinicians in the fight against cancer (104). Integrating EV-based approaches with current therapeutic modalities, may unlock new dimensions of precision medicine, tailored to the unique biological landscapes of individual patients.

Recent advances in targeting EVs present promising therapeutic avenues for BC. Genetic manipulation techniques, such as CRISPR/Cas9, allow for the disruption of genes involved in EV biogenesis, such as those encoding components of the ESCRT machinery, potentially reducing their pro-tumorigenic effects. RNA interference can downregulate oncogenic mRNAs within EVs, mitigating their impact on recipient cells (105). Introducing therapeutic genes into EVs can transform them into delivery vehicles for beneficial genetic material, potentially correcting oncogenic pathways in recipient cells. Pharmacological strategies include small molecule inhibitors like GW4869, which decrease EV release by targeting lipid rafts, and agents that block EV uptake by interfering with receptor interactions. Drugs that degrade or neutralize specific proteins or RNAs within EVs can reduce their pathogenic effects on BC. Immunotherapeutic approaches leverage monoclonal antibodies to neutralize tumor-derived EVs, while CAR T cells can be engineered to target specific EV antigens, enhancing immune clearance. Additionally, using EVs in vaccination strategies can elicit a strong anti-tumor immune response. These strategies, either alone or in combination, offer innovative pathways to improve BC treatment outcomes by specifically targeting EVs (106).

Exogenous and engineered EVs represent a frontier in cancer therapy, offering a platform for targeted therapeutic delivery. These EVs can be tailored through genetic editing or chemical modification to carry specific therapeutic agents, such as miRNAs, siRNAs, proteins, or small molecule drugs, thereby enhancing the precision and efficacy of targeted therapies (107). By incorporating specific ligands or antibodies on the EV surface, efficient targeting of particular cells or tissues can be achieved, while maintaining high biocompatibility and low immunogenicity. Engineered EVs are not only capable of drug delivery but also carry therapeutic biomarkers that modulate cellular signaling pathways to facilitate disease treatment. This feature enables them to play a crucial role in targeted therapy, providing more effective treatment strategies in BC.

While EV-based therapies show great promise, several critical challenges must be addressed for successful clinical translation. Recent discussions have highlighted the absence of clinical trials specifically focused on EV-based therapies for BC. This gap can be attributed to several challenges. The heterogeneity of EVs, particularly those derived from different TNBC subpopulations, complicates the development of standardized isolation and characterization protocols essential for consistent therapeutic outcomes (108). Current isolation methods often lack scalability and purity, necessitating innovations in purification technologies. Furthermore, achieving targeted delivery remains a challenge due to the natural distribution of EVs to multiple organs, which can lead to off-target effects. Engineering EVs with tissue-specific ligands could enhance precision but requires careful optimization to avoid immunogenicity. Additionally, the rapid clearance of EVs by the immune system limits their bioavailability, prompting the need for strategies to extend their half-life. Regulatory concerns also arise from the potential risks associated with EV cargo, needing rigorous preclinical safety profiling. The high cost and complexity of large-scale production further hinder clinical application, though automated systems and synthetic alternatives may offer solutions. Addressing these issues involves integrating advanced multi-omics approaches to better understand EV heterogeneity and developing combinatorial strategies that synergize EV therapies with existing treatments, such as immunotherapy, to overcome resistance and enhance efficacy in BC.

Additionally, the therapeutic application of EVs in BC faces challenges including low yield, heterogeneity, scalability, and quality control. Conventional isolation methods, such as ultracentrifugation, often result in low recovery and contamination, prompting the development of microfluidic and affinity-based approaches to improve efficiency. EV heterogeneity complicates their characterization, requiring advanced tools like single-particle analysis and standardized protocols. Scalability remains a major obstacle, as traditional 2D cultures are insufficient for large-scale production. Emerging solutions, such as 3D cultures and bioreactors, offer higher yields and continuous harvesting. Consistency and quality across production batches also require robust quality control systems and adherence to manufacturing standards. Cargo loading methods, such as electroporation, face limitations in preserving EV integrity, with alternative strategies like donor cell engineering showing promise. Stability during storage and transport is another critical issue, with optimization of cryopreservation and lyophilization techniques being explored. Addressing these hurdles through innovative technologies is essential for advancing EV-based therapies.

Future research on engineered EVs should prioritize systematic assessment of their safety and efficacy, alongside developing scalable production methods. In-depth exploration of the regulatory mechanisms governing EV biogenesis, secretion, and uptake will be crucial for optimizing their design and application, particularly in immune modulation and TME. By integrating these EVs with other therapeutic approaches, such as immune checkpoint inhibitors and targeted therapies, there is potential to enhance efficacy and overcome resistance. Additionally, analyzing patient-specific EV composition and function can lead to personalized treatment regimens, improving precision and therapeutic outcomes. Achieving these advancements will require interdisciplinary collaboration across biology, chemistry, engineering, and medicine to ensure successful translation from the laboratory to clinical practice.

## 11 Conclusion

Collectively, tumor-derived extracellular vesicles (EVs), enriched with diverse molecular cargo such as miRNAs, proteins, and non-coding RNAs, orchestrate complex interactions within the TME. Additionally, EVs impact immune cell behavior, promoting macrophage polarization, DCs maturation, and T-cell dysfunction, thereby facilitating tumor progression. Understanding these intricate mechanisms opens new avenues for therapeutic interventions, highlighting the potential of targeting EV-mediated pathways to enhance cancer treatment efficacy and patient outcomes.

## Glossary

EV-Apo: apoptotic TNBC cells
BHS: baohuoside I
BMDMs: bone marrow-derived macrophages
BC: breast cancer
CEBPD: CCAAT enhancer binding protein delta
CCL2: CC‐chemokine ligand 2
circRNAs: circular RNAs
CSF-1: colony stimulating factor-1
CDCP1: CUB-domain-containing protein 1
SOCS3: cytokine signaling 3
DAMPs: damage-associated molecular patterns
DCs: dendritic cells
ENPP1: ectonucleotide pyrophosphatase phosphodiesterase 1
ESCRT: endosomal sorting complexes required for transport
ETA: endothelin receptor A
EMT: epithelial-mesenchymal transition
EVs: extracellular vesicles
Gal3: galectin 3
Gal3BP: galectin 3 binding protein
GMPs: granulocyte-monocyte progenitors
HMGB-1: high-mobility group box 1
HIF1α: hypoxia-inducible factor-1α
ICAM1: intercellular adhesion molecule 1
IRF5: interferon regulatory factor 5
ILVs: intraluminal vesicles
RT-TEX: irradiated murine BC cells produced tumor exosomes
lncRNAs: long noncoding RNAs
MHC: major histocompatibility complex
MMP-13: matrix metalloproteinase 13
MPs: microparticles
miRNAs: micro-RNAs
mtDNA: mitochondrial DNA
moDCs: monocyte-derived dendritic cells
MDR: multidrug-resistant
MVBs: multivesicular bodies
MDSCs: myeloid-derived suppressor cells
NK: natural killer
NmU: neuromedin U
NETs: neutrophil extracellular traps
NETs: neutrophil extracellular traps
ncRNAs: non-coding RNAs
Ops: osteoprogenitor cells
PTX: paclitaxel
PD-L1: programmed death ligand-1
PIAS: protein inhibitor of activated STAT3
ROS: reactive oxygen species
TCR: T cell receptor
CTLs: T lymphocytes
TLR2: toll-like receptor 2
TNBC: triple-negative breast cancer
TME: tumor microenvironment
TAMs: tumor-associated macrophages
USP8: ubiquitin-specific peptidase 8
VEGF: vascular endothelial growth factor
XIST: X-inactive-specific transcript
